# The weekend effect on 28-day mortality in septic patients admitted to the ICU: A retrospective study from the MIMIC-IV database

**DOI:** 10.1371/journal.pone.0324288

**Published:** 2025-05-27

**Authors:** Jianmin Qu, Tingting Wang, Xiaoyu Zhou, Congcong Lv, Jiayi Chen, Shuhao Que

**Affiliations:** 1 Department of Intensive Care Unit, Tongxiang First People’s Hospital, Tongxiang, Zhejiang, P.R. China; 2 Department of Intensive Care Unit, The Second People’s Hospital of Liaocheng, Linqing, Shandong Province, P.R. China; 3 Hematopoietic Stem Cell Transplantation Center, State Key Laboratory of Experimental Hematology, National Clinical Research Center for Blood Diseases, Haihe Laboratory of Cell Ecosystem, Institute of Hematology and Blood Diseases Hospital, Chinese Academy of Medical Sciences and Peking Union Medical College, Tianjin, China; 4 Intensive Care Medicine Department, Affiliated Hangzhou First People’s Hospital, School of Medicine, Westlake University, Hangzhou, Zhejiang Province, China; 5 Zhejiang Chinese Medical University, The Second School of Clinical Medicine, Hangzhou City, Zhejiang Province, China; King Saud University College of Medicine, SAUDI ARABIA

## Abstract

**Objective:**

Numerous studies have shown that patients admitted on weekends or holidays have higher mortality rates and poorer prognoses than those admitted on weekdays. However, the specific impact of the weekend effect on patients with sepsis remains unclear and requires further investigation.

**Methods:**

This study included adult patients with sepsis who were first admitted to the ICU and stayed for ≥24 hours, using data from the Medical Information Mart for Intensive Care (MIMIC)-IV (version 2.2),with the data collection period from 2008 to 2019. Data on age, gender, type of ICU admission, vital signs, disease severity scores, and medications were collected, with patients categorized into weekend and weekday admission groups. The primary outcome was 28-day mortality, while secondary outcomes included 90-day mortality, hospital mortality, ICU mortality, and survival days without vasoactive drugs, ventilator, or ICU stay. COX regression analyses with propensity score matching (PSM) were employed to assess the impact of weekend admissions on the survival of septic patients in the ICU.

**Results:**

A total of 20,261 septic patients met the inclusion criteria, with 14,469 in the weekday group and 5,792 in the weekend group. The weekend admission group showed no statistically significant differences in 28-day mortality, hospital mortality, ICU mortality, survival days without vasoactive drugs, survival days without ventilator, survival days without ICU, and length of ICU stay compared to the weekday group. Subgroup analyses for 28-day mortality revealed that key baseline characteristics such as gender, age, BMI, race, ICU type, hypertension, diabetes mellitus, and SOFA score did not independently influence the prognosis of patients with sepsis based on weekend admission.

**Conclusion:**

The study found no significant weekend effect on the prognosis of septic patients admitted to the ICU, based on both univariate and multivariate analyses.

## Background

The weekend effect, where patients admitted on weekends or holidays experience higher mortality or poorer outcomes than those admitted on weekdays, has garnered significant academic attention [1]^.^ This phenomenon has been observed across various acute conditions, including acute myocardial infarction, stroke, and trauma [2–4]. The reduced healthcare staff on weekends, driven by cost pressures and market realities, is a potential contributor to this effect [5].

Studies have consistently shown that ICU admissions on weekends are associated with significantly higher in-hospital mortality rates compared to weekday admissions [6–8]. Sepsis, a prevalent and life-threatening condition in ICUs characterized by organ dysfunction due to a dysregulated host response to infection [9], has high morbidity and mortality rates. It is estimated that over 30 million people suffer from sepsis annually, with more than 5 million resulting in death worldwide [10].

While the weekend effect on ICU patients has been extensively studied, its specific impact on sepsis patients remains underexplored. This study aims to investigate the significance and potential mechanisms of the weekend effect in ICU sepsis patients and propose appropriate improvement measures.

## Methodologies

### Data sources

This study utilized the Medical Information Mart for Intensive Care (MIMIC)-IV (version 2.2) database, which contains information on 53,150 ICU patients from 2008 to 2019 [11,12]. The database was approved for research by the institutional review boards of the Massachusetts Institute of Technology (No. 0403000206) and Beth Israel Deaconess Medical Center (2001-P-001699/14). Ethical regulations related to data research were followed, and all participants passed the necessary ethical exams and were granted access to the MIMIC database.

### Study population

The study included:1.Adult sepsis patients (18 years or older) who were first admitted to the ICU in MIMIC-IV and stayed for at least 24 hours.2.Only patients with a single ICU admission were considered; those with multiple ICU admissions were excluded.3.Sepsis was defined as the presence of an infection and an increase in SOFA score by at least 2 points [13,14].

### Study process

**Fig 1:**
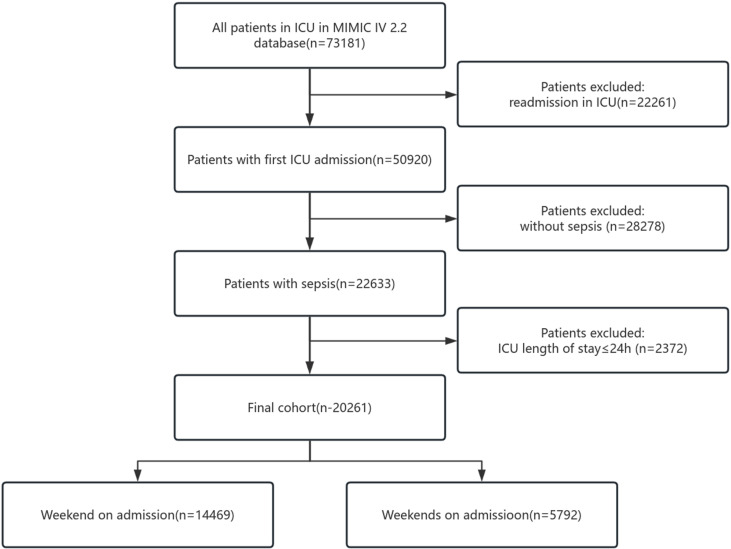
Flowchart of the study cohort. ICU: Intensive Care Unit, MIMIC-IV: Medical Information Mart for Intensive Care-IV.

Out of 73,181 patients admitted to the ICU from the MIMIC-IV 2.2 database, 22,261 patients with repeat admissions, 28,287 non-sepsis patients, and 2,372 patients with ICU stays of less than 24 hours were excluded. A total of 20,261 patients met the inclusion criteria. These patients were categorized based on the time of admission into weekday group (0:00 a.m. Monday to 11:59 p.m. Friday) (n = 14,469) and weekend group (0:00 a.m. Saturday to 11:59 p.m. Sunday) (n = 5,792). The detailed flow is illustrated in Fig 1.

### Data collection

Patients with sepsis included in this study were obtained from the MIMIC-IV version 2.2 database. Data collected included age, gender, race, body mass index (BMI), marital status, insurance status, type of ICU admission (coronary care unit (CCU), medical intensive care unit (MICU), neurosurgical intensive care unit (NICU), and surgical intensive care unit (SICU)), whether elective surgery was performed, duration of ICU stay, vital signs (temperature, heart rate, respiratory rate, mean arterial pressure (MAP), pulse oximetry (SPO2)), laboratory parameters (e.g., white blood cell count, hemoglobin), co-morbidities (e.g., hypertension, coronary artery disease, diabetes mellitus), disease severity scores (e.g., Simplified Acute Physiological Fractionation Score (SOFA), Oxford Acute Severity of Illness Score (OASIS), Simplified Acute Physiological Score (SAPS II)), and day 1 medications and treatment regimens (invasive mechanical ventilation (IMV), renal replacement therapy (RRT), vasoactive medications, antibiotics, fluid intake).

### Main outcome

The primary outcome of this study was 28-day mortality in septic patients admitted to the ICU. Secondary outcomes included 90-day mortality, hospital mortality, ICU mortality, and length of ICU stay.

### Statistical methods

All patients were analyzed descriptively. Categorical variables were expressed as numbers and percentages, while continuous variables were expressed as mean and standard deviation (SD) for normally distributed data or median and interquartile range for skewed data. The chi-square test, t-test, and Kruskal-Wallis test were used to compare categorical variables, normally distributed, and non-normally distributed continuous variables, respectively. COX regression analyses with propensity score matching (PSM) were employed to assess whether weekend stays affected survival in ICU sepsis patients. All analyses were performed using the R statistical package (version 4.2.3) and Stata (version 17.0), with a p-value < 0.05 in two-tailed tests considered statistically significant.

## Results

### Baseline characteristics

Table 1 presents the baseline characteristics of the 20,261 patients enrolled in the study. The mean age was 65.9 ± 16.3 years, with 58.1% male. Over half of the patients were admitted to the ICU on an emergency basis, with a median admission time of 13:00. Patients were admitted to various ICU units: CCU, MICU, NICU, and SICU, with MICU accounting for nearly 40% and NICU for only 3.6%. The patients’ vital signs were generally stable, with an average heart rate of 106 bpm and pulse oximetry above 90%. Hypertension was present in 64.5% of patients and coronary artery disease in 31.8% (n = 6435). Regarding treatments, over 80% received antibiotics, and 57.3% (n = 11,608) underwent invasive mechanical ventilation. The 28-day mortality rate was 17.7%, and the median ICU stay was 3.1 days. Notably, factors such as age, SPO2, and COPD prevalence differed significantly between weekday and weekend admissions, necessitating further PSM analysis. Primary outcome indicators remained stable post-PSM.

**Table 1:** Baseline characteristics of participants.

Variables	Total (n = 20261)	Weekends (n = 5792)	Weekdays (n = 14469)	*P*-value
**General characteristics**				
Age (years)	65.9 ± 16.3	65.5 ± 16.7	66.0 ± 16.1	**0.048**
Gender, n (%)				0.999
Female	8483 (41.9)	2425 (41.9)	6058 (41.9)	
Male	11778 (58.1)	3367 (58.1)	8411 (58.1)	
Ethnicity, n (%)				0.474
Asian	568 (2.8)	162 (2.8)	406 (2.8)	
Black	1581 (7.8)	465 (8.0)	1116 (7.7)	
Other	4518 (22.3)	1325 (22.9)	3193 (22.1)	
White	13594 (67.1)	3840 (66.3)	9754 (67.4)	
BMI (kg/m^2^)	29.2 ± 7.6	29.0 ± 7.6	29.3 ± 7.7	0.053
Marital status, n (%)				0.235
Single	11131 (54.9)	3220 (55.6)	7911 (54.7)	
Married	9130 (45.1)	2572 (44.4)	6558 (45.3)	
Insurance, n (%)				0.869
Medicaid	1435 (7.1)	406 (7.0)	1029 (7.1)	
Medicare	9341 (46.1)	2658 (45.9)	6683 (46.2)	
Other	9485 (46.8)	2728 (47.1)	6757 (46.7)	
Admissions care unit, n (%)				0.966
CCU	6342 (31.3)	1800 (31.1)	4542 (31.4)	
MICU	8049 (39.7)	2307 (39.8)	5742 (39.7)	
NICU	739 (3.6)	209 (3.6)	530 (3.7)	
SICU	5131 (25.3)	1476 (25.5)	3655 (25.3)	
Admission type, n (%)				0.831
Elective	1013 (5.0)	276 (4.8)	737 (5.1)	
EMER	10738 (53.0)	3071 (53.0)	7667 (53.0)	
Observation	1838 (9.1)	535 (9.2)	1303 (9)	
Surgical	2285 (11.3)	644 (11.1)	1641 (11.3)	
Urgent	4387 (21.7)	1266 (21.9)	3121 (21.6)	
Elective surgery, n (%)	923 (4.6)	252 (4.4)	671 (4.6)	0.377
ICU admission time (hours)	13.0 (8.0, 18.0)	13.0 (8.0, 18.0)	13.0 (8.0, 18.0)	0.955
**Vital signs**				
Body temperature (°C)	37.5 ± 0.8	37.5 ± 0.8	37.5 ± 0.8	0.571
Heart Rate (bpm)	106.0 ± 20.9	106.3 ± 21.0	105.8 ± 20.9	0.102
Respiratory Rate (bpm)	28.6 ± 6.4	28.7 ± 6.4	28.6 ± 6.4	0.267
MAP (mmHg)	56.5 ± 13.3	56.4 ± 13.5	56.5 ± 13.2	0.884
SPO_2_ (%)	91.5 ± 6.4	91.4 ± 6.8	91.6 ± 6.3	0.047
**Laboratory parameters**				
White blood cell (×10^9^/L)	13.3 (9.5, 18.0)	13.3 (9.4, 18.0)	13.3 (9.5, 18.0)	0.971
Hemoglobin (g/dL)	9.8 ± 2.2	9.8 ± 2.2	9.8 ± 2.2	0.877
Platelet (×10^9^/L)	159.0 (111.0,224.0)	160.0 (110.0,227.0)	159.0 (111.0,223.0)	0.426
Potential of hydrogen	7.3 ± 0.1	7.3 ± 0.1	7.3 ± 0.1	0.295
PaO_2_ (mmHg)	90.0 (70.0, 121.0)	91.0 (71.0, 123.0)	90.0 (70.0, 120.0)	0.336
PaCO_2_ (mmHg)	46.9 ± 13.4	46.8 ± 13.4	47.0 ± 13.4	0.371
Lactate (mmol/L)	2.9 ± 2.4	2.9 ± 2.4	2.9 ± 2.4	0.284
Glucose (mg/dL)	167.0 (134.0, 213.0)	166.0 (133.0, 211.0)	167.5 (134.0, 214.0)	0.256
Blood bicarbonate (mEq/L)	21.6 ± 4.7	21.6 ± 4.7	21.6 ± 4.7	0.803
Serum potassium (mEq/L)	4.5 (4.0, 5.1)	4.5 (4.0, 5.1)	4.5 (4.1, 5.1)	0.692
Serum natrium (mEq/L)	140.0 ± 8.2	140.0 ± 5.3	139.9 ± 9.1	0.562
Blood chlorine (mEq/L)	106.8 ± 7.0	106.8 ± 6.8	106.8 ± 7.1	0.519
Blood calcium (mg/dL)	8.4 ± 0.9	8.4 ± 0.9	8.4 ± 0.9	0.680
BUN (mg/dL)	21.0 (15.0, 35.0)	21.0 (14.0, 36.0)	21.0 (15.0, 35.0)	0.540
Serum creatinine (mg/dL)	1.10 (0.8, 1.7)	1.10 (0.8, 1.7)	1.10 (0.8, 1.7)	0.313
APTT (sec)	32.8 (28.4, 42.3)	32.7 (28.4, 41.8)	32.8 (28.4, 42.4)	0.628
**Comorbidities**				
CHF, n (%)	5537 (27.3)	1552 (26.8)	3985 (27.5)	0.134
Hypertension, n (%)	13071 (64.5)	3721 (64.2)	9350 (64.6)	0.232
Diabetes, n (%)	5804 (28.6)	1627 (28.1)	4177 (28.9)	0.131
COPD, n (%)	2931 (14.5)	776 (13.4)	2155 (14.9)	0.006
CAD, n (%)	6435 (31.8)	1818 (31.4)	4617 (31.9)	0.203
Stroke, n (%)	2264 (11.2)	670 (11.6)	1594 (11.0)	0.124
**Disease severity scores**				
APSIII score	49.2 ± 21.8	49.1 ± 21.3	49.2 ± 21.9	0.685
SAPSII score	40.0 ± 14.0	39.8 ± 14.0	40.0 ± 14.1	0.368
SOFA score	5.8 ± 3.4	5.8 ± 3.4	5.9 ± 3.5	0.745
Charlson score	5.1 ± 2.9	5.0 ± 2.9	5.1 ± 3.0	**0.013**
mNUTRIC score	4.1 ± 1.9	4.0 ± 2.0	4.1 ± 1.9	**0.017**
APACHE II score	19.4 ± 7.2	19.2 ± 7.2	19.4 ± 7.2	**0.019**
**Medication or procedures**				
IMV, n (%)	11608 (57.3)	3328 (57.5)	8280 (57.2)	0.762
RRT, n (%)	615 (3.0)	169 (2.9)	446 (3.1)	0.537
Vasoactive drug, n (%)	9727 (48.0)	2805 (48.4)	6922 (47.8)	0.449
Antibiotic, n (%)	17596 (86.8)	5054 (87.3)	12542 (86.7)	0.273
Fluid input (ml)	3191.4 (1849.6, 4807.4)	3229.3 (1887.1, 4834.6)	3168.6 (1834.8, 4796.8)	0.303
**Outcomes**				
28-day mortality, n (%)	3590 (17.7)	1007 (17.4)	2583 (17.9)	0.433
90-day mortality, n (%)	4840 (23.9)	1363 (23.5)	3477 (24)	0.452
Hospital mortality, n (%)	2942 (14.5)	829 (14.3)	2113 (14.6)	0.596
ICU mortality, n (%)	2076 (10.2)	580 (10.0)	1496 (10.3)	0.490
Vasoactive drug free 28 day(d)	27.8 (24.3, 28.0)	27.8 (24.4, 28.0)	27.8 (24.2, 28.0)	0.537
Ventilation free 28 day (d)	27.5 (23.1, 28.0)	27.5 (23.3, 28.0)	27.5 (23.1, 28.0)	0.388
ICU free 28 day (d)	24.1 (14.3, 26.0)	24.1 (14.8, 26.1)	24.1 (14.2, 26.0)	0.308
Length of ICU (d)	3.1 (1.8, 6.4)	3.1 (1.8, 6.3)	3.1 (1.9, 6.4)	0.346

BMI: body mass index, CCU: Coronary Care Unit, MICU: Medical Intensive Care Unit, NICU: Neuro Surgical Intensive Care Unit, SICU: Surgical Intensive Care Unit, ICU: Intensive Care Unit, MAP: mean arterial pressure, SPO2: Pulse Oxygen Saturation,PaO2: partial pressure of oxygen in arterial blood, PaCO2: carbon dioxide partial pressure, BUN: blood urea nitrogen, APTT: activated partial thromboplastin time, CHF: congestive heart failure, COPD: chronic obstructive pulmonary disease, CAD: Coronary Artery Disease, APSIII: Acute Physiology Score III, SAPSII: simplified acute physiologic score II, SOFA: Sequential Organ Failure Assessment, mNUTRIC: modified nutrition risk in critically ill, APACHE II: acute physiology and chronic health evaluation, IMV: invasive mechanical ventilation, RRT: renal replacement treatment.

### Primary outcome

Four multifactorial Cox regression models were constructed stepwise to examine 28-day mortality in sepsis patients, comparing weekend versus weekday admissions. These models incorporated basic characteristics, vital signs within 24 hours of ICU admission, laboratory tests, complications, disease severity scores, and treatment modalities (see Table 2). None of the models demonstrated statistically significant differences. Considering that our study excluded patients with ICU stays of less than 24 hours, we conducted a separate statistical analysis for this subgroup. A Cox regression model was constructed for patients with ICU length of stay less than 24 hours, and no statistically significant differences were found between the groups (see S1 Table). After incorporating additional variables such as septic shock, time of initiating antibiotics and obtaining blood cultures, and delays in obtaining blood cultures and initiating antibiotics, a repeated Cox regression analysis similarly indicated no statistically significant differences in outcomes between weekend and weekday ICU admissions (see S2 Table). Likewise, a Cox regression analysis was performed for patients with ICU stays longer than 72 hours, and consistent results were observed—no significant difference in prognosis was found between the weekend and weekday ICU admission groups (see S3 and S4 Tables).Adjusting for various statistical methods, including crude analysis, multivariate analysis, propensity score adjustment, matching, inverse probability weighting (IPTW), standardized mortality weighting (SMRW), propensity adjustment (PA), and overall analysis (Ow), the weekend admission group did not show a significant difference compared to the weekday admission group (see S1 Fig).

**Table 2:** Multivariable Cox regression analysis of 28-day mortality for sepsis patients admitted on weekends.

Variable	N. total	N. Event%	Model I		Model II		Model III		Model IV	
			HR (95%CI)	*P*-value	HR (95%CI)	*P*-value	HR (95%CI)	*P*-value	HR (95%CI)	*P*-value
Weekdays	14469	2583 (17.9)	1.00 (Ref)		1.00 (Ref)		1.00 (Ref)		1.00 (Ref)	
Weekends	5792	1007 (17.4)	0.97 (0.90 ~ 1.04)	0.427	0.96 (0.90 ~ 1.04)	0.326	0.97 (0.90 ~ 1.04)	0.422	1.00 (0.93 ~ 1.07)	0.962

**Note:** Model I: Unadjusted. Model II: Adjusted for gender, age, ethnicity, BMI, marital status, insurance, admissions care unit, admission type, elective surgery, ICU admission time (hours), body temperature, heart rate, respiratory rate, and MAP, SPO2. Model III: Model II plus white blood cell, hemoglobin, platelets, potential of hydrogen, PaO2, PaCO2, lactate, blood bicarbonate, glucose, blood urea nitrogen, serum creatinine, serum natrium, blood chlorine, blood calcium, APTT. Model IV: Model III plus CHF, hypertension, diabetes, COPD, CAD, stroke, APSIII, SAPSII, SOFA, charlson, APACHE II, mNUTRIC, IMV, vasoactive drug, RRT, antibiotic, fluid input on first day. HR: hazard ratio, CI: confidence interval, Ref: reference, BMI: body mass index, MAP: mean arterial pressure, SPO2: Pulse Oxygen Saturation, PaO2: partial pressure of oxygen in arterial blood, PaCO2: carbon dioxide partial pressure, CHF: congestive heart failure, APTT: activated partial thromboplastin time, COPD: chronic obstructive pulmonary disease, CAD: Coronary Artery Disease, APSIII: Acute Physiology Score III, SAPSII: Simplified Acute Physiologic Score II, SOFA: Sequential Organ Failure Assessment, APACHE II: acute physiology and chronic health evaluation, mNUTRIC: Modified Nutrition Risk in Critically ill, IMV: invasive mechanical ventilation, RRT: renal replacement treatment.

### Secondary outcomes

Multifactorial Cox regression models were constructed to evaluate 90-day mortality, hospital mortality, and ICU mortality as secondary outcomes. No statistically significant differences were observed between weekend and weekday admissions for any of these secondary outcomes (see Table 3).

**Table 3:** Multivariate Cox regression analysis of outcomes for sepsis patients admitted on weekends.

Variable	N. Total	N. Event%	Model I		Model II		Model III		Model IV	
			HR (95%CI)	*P*-value	HR (95%CI)	*P*-value	HR (95%CI)	*P*-value	HR (95%CI)	*P*-value
**90-day mortality**										
Weekdays	14469	3477 (24.0)	1.00 (Ref)		1.00 (Ref)		1.00 (Ref)		1.00 (Ref)	
Weekends	5792	1363 (23.5)	0.98 (0.92 ~ 1.04)	0.441	0.94 (0.85 ~ 1.04)	0.251	0.93 (0.83 ~ 1.03)	0.167	0.94 (0.85 ~ 1.05)	0.275
**ICU mortality**										
Weekdays	14469	1496 (10.3)	1.00 (Ref)		1.00 (Ref)		1.00 (Ref)		1.00 (Ref)	
Weekends	5792	580 (10.0)	0.97 (0.88 ~ 1.06)	0.481	0.90 (0.77 ~ 1.06)	0.214	0.91 (0.78 ~ 1.07)	0.258	0.90 (0.76 ~ 1.05)	0.181
**Hospital mortality**										
Weekdays	14469	2113 (14.6)	1.00 (Ref)		1.00 (Ref)		1.00 (Ref)		1.00 (Ref)	
Weekends	5792	829 (14.3)	1.00 (0.92 ~ 1.08)	0.933	0.89 (0.78 ~ 1.02)	0.097	0.90 (0.78 ~ 1.03)	0.114	0.90 (0.79 ~ 1.04)	0.148

**Note:** Model I: Unadjusted. Model II: Adjusted for gender, age, ethnicity, BMI, marital status, insurance, admissions care unit, admission type, elective surgery, ICU admission time (hours), body temperature, heart rate, respiratory rate, and MAP, SPO2. Model III: Model II plus white blood cell, hemoglobin, platelets, potential of hydrogen, PaO2, PaCO2, lactate, blood bicarbonate, glucose, blood urea nitrogen, serum creatinine, serum natrium, blood chlorine, blood calcium, APTT. Model IV: Model III plus CHF, hypertension, diabetes, COPD, CAD, stroke, APSIII, SAPSII, SOFA, charlson, APACHE II, mNUTRIC, IMV, vasoactive drug, RRT, antibiotic, fluid input on first day. HR: hazard ratio, CI: confidence interval, Ref: reference, BMI: body mass index, MAP: mean arterial pressure, SPO2: Pulse Oxygen Saturation, PaO2: partial pressure of oxygen in arterial blood, PaCO2: carbon dioxide partial pressure, CHF: congestive heart failure, APTT: activated partial thromboplastin time, COPD: chronic obstructive pulmonary disease, CAD: Coronary Artery Disease, APSIII: Acute Physiology Score III, SAPSII: Simplified Acute Physiologic Score II, SOFA: Sequential Organ Failure Assessment, APACHE II: acute physiology and chronic health evaluation, mNUTRIC: Modified Nutrition Risk in Critically ill, IMV: invasive mechanical ventilation, RRT: renal replacement treatment.

### Subgroup analysis

**Fig 2:**
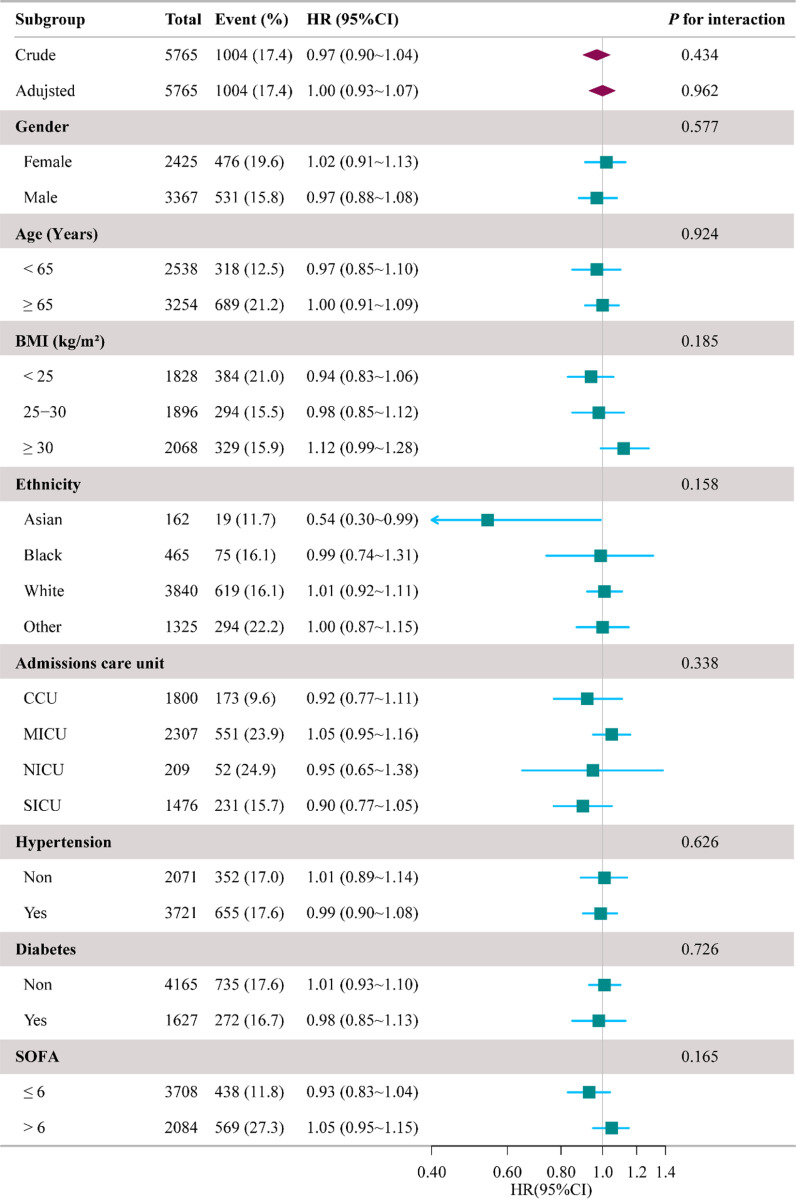
Subgroup analysis forest plot illustrating the hazard ratios (HRs) for 28-day mortality associated with weekend, stratified by key baseline characteristics. HR: hazard ratio, CI: confidence interval, BMI: body mass index, CCU: Coronary Care Unit, MICU: Medical Intensive Care Unit, NICU: Neuro Surgical Intensive Care Unit, SICU: Surgical Intensive Care Unit, SOFA: Sequential Organ Failure Assessment.

The 28-day mortality rates were stratified by key baseline characteristics, including age (>65 years), sex (male, female), race (Asian, Black, White, other), type of ICU (CCU, MICU, NICU, SICU), presence of hypertension, diabetes mellitus, and SOFA scores (>6 points) (see Fig 2).

### Propensity score analysis

Differences in 28-day mortality between weekend and weekday ICU admissions were matched using propensity scores. The results remained stable and showed no statistically significant differences after matching (see S1 Fig).

## Discussion

In this study, 20,261 patients with sepsis were categorized into a weekday group (n = 14,469) and a weekend group (n = 5,792). Multifactorial Cox regression analyses were conducted with 28-day mortality as the primary outcome, and 90-day mortality, hospital mortality, and ICU mortality as secondary outcomes. No statistically significant differences were found between the two groups. After propensity score matching (PSM), the results indicated that the grouping variable did not influence sepsis prognosis. These findings remained consistent after adjusting statistical methods, refining the study population data, and stratifying the model. Key baseline characteristics, including gender, age, BMI, race, ICU type, hypertension, diabetes, and SOFA score, also did not impact the prognosis of patients admitted on weekends.

A large retrospective study [6] demonstrated that ICU mortality was higher on weekends (11.3%) compared to weekdays (7.0%) (OR=1.70; 95% CI: 1.55–1.85), potentially due to increased severity of cases admitted on weekends. However, after adjusting for confounders, no significant association was found between weekend admissions and inpatient mortality (OR=1.06; 95% CI: 0.95–1.17), aligning with our study’s findings. This might be attributed to effective weekend medical staffing. Hospital resources and staffing levels have been shown to impact mortality rates [15], and shift systems may also play a role [16].

A cohort study [17] involving 2093 patients categorized into weekday (31%), weekday evening (35%), and weekend groups (34%) found no significant difference in hospital mortality rates among the groups (36%, 36%, and 37%, p = 0.90). This supports the notion that subsequent daily care has a greater impact on patient prognosis than initial symptomatic management, which is consistent across all times [18,19].

Conversely, another retrospective study [20] reported increased mortality for weekend admissions, even after adjusting for disease severity (OR=1.05; 95% CI: 1.04–1.06), possibly due to longer hospital stays at lower costs. The absence of a significant difference in ICU stay duration in our baseline results may explain our negative findings. Other studies [21,22] similarly indicated that weekend hospitalization increased mortality, often linked to disease severity.

Interestingly, one study [23] found that off-hours admissions had slightly lower in-hospital mortality rates than weekday admissions (adjusted OR = 0.93; 95% CI: 0.87–0.98), potentially because weekday medical staff handle more tasks that could interfere with patient management and care quality [24,25].

In conclusion, our findings indicate that weekend ICU admissions do not significantly impact the 28-day mortality rate of sepsis patients after adjusting for confounding factors such as disease severity. This outcome is likely due to the ICU shift system, which ensures adequate healthcare resource allocation around the clock [19]. The Surviving Sepsis Campaign is sponsored by the European Society of Intensive Care Medicine and the American Society of Critical Care Medicine co-launched it in 2004 [26]. The two associations define and analyze sepsis and issue medical guidelines to better address the challenges posed by sepsis. After 20 years of continuous efforts [27], the identification and treatment of sepsis has been very efficient, which is one of the possible reasons why weekend effects are not significantly different in patients with sepsis in ICU.

Other related studies have found that increased mortality in weekend patients may be associated with abnormalities in various common hematologic test results, particularly lymphocyte counts, sodium concentrations, and urea concentrations [28]. In the present study, baseline laboratory measurements were not significantly different between the two groups, potentially explaining why mortality was not significantly higher in the weekend group. Another study found that ICU admission time could affect ICU mortality, with admissions between 00:00–03:59 (HR: 1.17; 95% CI: 1.08–1.28) and 04:00–07:59 (HR: 1.16; 95% CI: 1.05–1.29) presenting higher risks [29]. However, our study showed that the median ICU admission time was 13:00 for both groups, with no statistical difference.

Furthermore, the type of ICU admission (CCU, MICU, NICU, and SICU) did not independently affect 28-day mortality in patients with sepsis (P = 0.338). Subgroup analysis from another study indicated higher adjusted hospital mortality for weekend ICU admissions in surgical units (OR=1.23; 95% CI: 1.03–1.48), but not in other medical or multispecialty ICUs, possibly due to varying care levels and the poor performance of APACHE II scores for surgical patients [6,30]. The diverse critical care scores used in our study mitigate these confounding factors.

The SOFA score effectively characterizes organ dysfunction/failure in critically ill patients, with regular scoring aiding in patient condition monitoring and understanding disease progression [31]. A study found that off-hours discharges had higher mean SOFA scores and were linked to increased in-hospital mortality, though off-hours discharges were not associated with organ dysfunction after multifactorial analysis [32]. Neurological and renal dysfunction, as measured by the SOFA score, are associated with increased in-hospital mortality [33]. However, in our study, there was no significant difference in SOFA scores between weekend and weekday admissions, nor did it affect 28-day mortality in septic patients.

Certainly, the ICU nurse-to-physician ratio is a critical factor influencing patient outcomes, particularly in assessing the impact of the ICU environment on patient mortality. A systematic review and meta-analysis, including one randomized controlled trial and 17 non-randomized studies, found no significant association between nighttime intensivist staffing and patient mortality [34]. Additionally, a survey of academic ICUs in the United States reported that approximately one-third of ICUs have implemented 24-hour in-hospital intensivist coverage [35].Some scholars have proposed that a 24/7 intensivist staffing model may offer certain advantages, such as reducing mortality, decreasing complications, shortening hospital stays, lowering healthcare costs, and enhancing physician satisfaction. However, the evidence supporting these benefits remains inconclusive [36]. Moreover, studies have indicated that ICU staffing and workload tend to be more stable compared to emergency departments, primarily due to the well-defined roles of ICU personnel, the clear patient population characteristics, and the fact that patient numbers are generally constrained by bed capacity, resulting in relatively lower fluctuations [37]. A cross-sectional study published in 2024 investigated the relationship between ICU staffing ratios and patient outcomes in the United States. The findings demonstrated no significant association between intensivist staffing ratios, shift models, and ICU patient mortality or length of stay. However, the study lacked a detailed description of specific ICU shift models, which limits the generalizability of its conclusions [38]. Notably, multicenter studies have indicated that nighttime intensivist staffing may be associated with reduced in-hospital mortality for patients admitted to ICUs with low-intensity daytime staffing. However, no additional benefits have been observed for patients in ICUs with high-intensity daytime staffing [39].The data used in this study were obtained from the MIMIC database, which includes clinical information on ICU patients from Beth Israel Deaconess Medical Center (BIDMC) in the United States. Although specific staffing ratios for the BIDMC ICU have not been publicly disclosed, as one of the leading medical institutions in the country, its ICU is likely to adhere to high or even more stringent standards of care. Based on this, we hypothesize that in a high-standard medical environment, the 28-day mortality rate of patients with sepsis may not differ significantly between weekend and weekday ICU admissions.

In summary, baseline characteristics such as gender, age, BMI, race, ICU type, hypertension, diabetes, and SOFA score do not influence 28-day mortality in sepsis patients admitted on weekends. Further detailed studies are necessary to explore these conclusions.

## Conclusion

This study demonstrated that the weekend effect did not significantly influence the prognosis of sepsis patients in ICUs. Factors such as the type of ICU, admission timing, and effective daily management likely contribute to this finding. The scientific rationalization of shift systems, adequate medical resources, and consistently high levels of care are integral in modern ICUs. Further research is necessary to explore additional factors that could optimize the treatment and management of sepsis patients, ultimately improving their survival rates.

### Limitations

This study has several limitations. As a retrospective analysis based on the MIMIC-IV database, it may suffer from missing or inaccurately recorded variables. Despite adjusting for confounders using propensity score matching and multifactorial Cox regression modeling, unmeasured confounding variables might still affect the results. Additionally, being a single-center study, its findings are specific to ICUs in the United States and may not be generalizable to other countries. Variations in hospital duty systems and management practices further limit the applicability of the results. Our research team plans to conduct multi-center data validation to enhance the diversity and completeness of the data. Future studies may adopt a prospective design to collect real-time patient data, thereby reducing the impact of unmeasured confounding variables.

## Supporting information

S1 Table Multivariable cox regression analysis of 28-day mortality in sepsis patients admitted on weekends: ICU length of stay <24 hours. (DOCX)

S2 Table Multivariable cox regression analysis of 28-day mortality in sepsis patients admitted on weekends: ICU length of stay <24 hours. (DOCX)

S3 Table Multivariate cox regression analysis of 28-day mortality in sepsis patients admitted on weekends with completed data. (DOCX)

S4 Table Relationship of the weekend effect to 28-day mortality in septic shock subgroups. (DOCX)

S1 Fig Forest plot displays the hazard ratios (HRs) for 28-day mortality in the weekends group, as determined by several analytical methods including crude analysis, multivariable analysis, propensity score adjustment, matching, inverse-probability-of-treatment weighted(IPTW), Standardized Mortality Ratio Weighted (SMRW), propensity adjusted (PA), and overall (Ow) analysis. (TIF)

S1 File Dataset. (XLSX)
