# Supplementary material for: The weekend effect on 28-day mortality in septic patients admitted to the ICU: A retrospective study from the MIMIC-IV database
Source: PLoS One. 2025 May 27;20(5):e0324288. doi: 10.1371/journal.pone.0324288 (PMC12111631; doi:10.1371/journal.pone.0324288)
Supplement: S2 Table — (DOCX) [file pone.0324288.s002.docx]

Table S2 Multivariable Cox Regression Analysis of 28-Day Mortality in Sepsis Patients Admitted on Weekends: ICU Length of Stay <24 Hours

| **variable** | **n.total** | **n.event%** | **Model I** | | **Model II** | | **Model III** | | **Model IV** | |
| --- | --- | --- | --- | --- | --- | --- | --- | --- | --- | --- |
|  |  |  | HR(95%CI) | *P*-value | HR(95%CI) | *P*-value | HR(95%CI) | P-value | HR(95%CI) | *P*-value |
| Weekdays | 669 | 173 (25.9) | 1.00(Ref) |  | 1.00(Ref) |  | 1.00(Ref) |  | 1.00(Ref) |  |
| Weekends | 1703 | 414 (24.3) | 0.93 (0.78~1.11) | 0.395 | 1.04 (0.86~1.24) | 0.709 | 1.02 (0.85~1.22) | 0.856 | 1.02 (0.84~1.24) | 0.842 |

**Note**

Model I: Unadjusted.

Model II: Adjusted for gender, age, ethnicity, BMI, marital status, insurance, admissions care unit, admission type, elective surgery, ICU admission time (hours), body temperature, heart rate, respiratory rate, and MAP, SPO2.

Model III: Model II plus white blood cell, hemoglobin, platelets, potential of hydrogen, PaO_2_, PaCO_2_, lactate, blood bicarbonate, glucose, blood urea nitrogen, serum creatinine, serum natrium, blood chlorine, blood calcium, APTT.

Model IV: Model III plus CHF, hypertension, diabetes, COPD, CAD, stroke, APSIII, SAPSII, SOFA, charlson, APACHE II, mNUTRIC, IMV, vasoactive drug, RRT, antibiotics, fluid input on first day, septic shock, time of initiating antibiotics and obtaining blood cultures, delays in obtaining blood cultures and initiating antibiotics

**Abbreviations:**

ICU: Intensive Care Unit, HR: hazard ratio, CI: confidence interval, Ref: reference, BMI: body mass index, MAP: mean arterial pressure, SPO_2_: Pulse Oxygen Saturation, PaO_2_: partial pressure of oxygen in arterial blood, PaCO_2_: carbon dioxide partial pressure, CHF: congestive heart failure, APTT: activated partial thromboplastin time, COPD: chronic obstructive pulmonary disease, CAD: Coronary Artery Disease, APSIII: Acute Physiology

Score III, SAPSII: Simplified Acute Physiologic Score II, SOFA: Sequential Organ Failure Assessment, APACHE II: acute physiology and chronic health evaluation, mNUTRIC: Modified Nutrition Risk in Critically ill, IMV: invasive mechanical ventilation, RRT: renal replacement treatment.
