# Supplementary material for: The weekend effect on 28-day mortality in septic patients admitted to the ICU: A retrospective study from the MIMIC-IV database
Source: PLoS One. 2025 May 27;20(5):e0324288. doi: 10.1371/journal.pone.0324288 (PMC12111631; doi:10.1371/journal.pone.0324288)
Supplement: S4 Table — (DOCX) [file pone.0324288.s004.docx]

**Table S4.** Relationship of the Weekend Effect to 28-Day Mortality in Septic Shock Subgroups

| **Septic shock** | **Variable** | **n.total** | **n.event %** | **HR (95% CI)** | **P value** | **P for interaction** |
| --- | --- | --- | --- | --- | --- | --- |
| No | Weekends | 4885 | 706 (14.5) | 1(Ref) |  | 0.261 |
|  | Weekdays | 12083 | 1758 (14.5) | 1.04 (0.95~1.14) | 0.381 |  |
| Yes | Weekends | 907 | 301 (33.2) | 1(Ref) |  |  |
|  | Weekdays | 2386 | 825 (34.6) | 0.95 (0.83~1.09) | 0.46 |  |

Note: Adjusted covariates included in Model IV.

**Abbreviations:**

HR: hazard ratio, CI: confidence interval, Ref: reference
