# Supplementary figures and images for: The weekend effect on 28-day mortality in septic patients admitted to the ICU: A retrospective study from the MIMIC-IV database
Source: PLoS One. 2025 May 27;20(5):e0324288. doi: 10.1371/journal.pone.0324288 (PMC12111631; doi:10.1371/journal.pone.0324288)

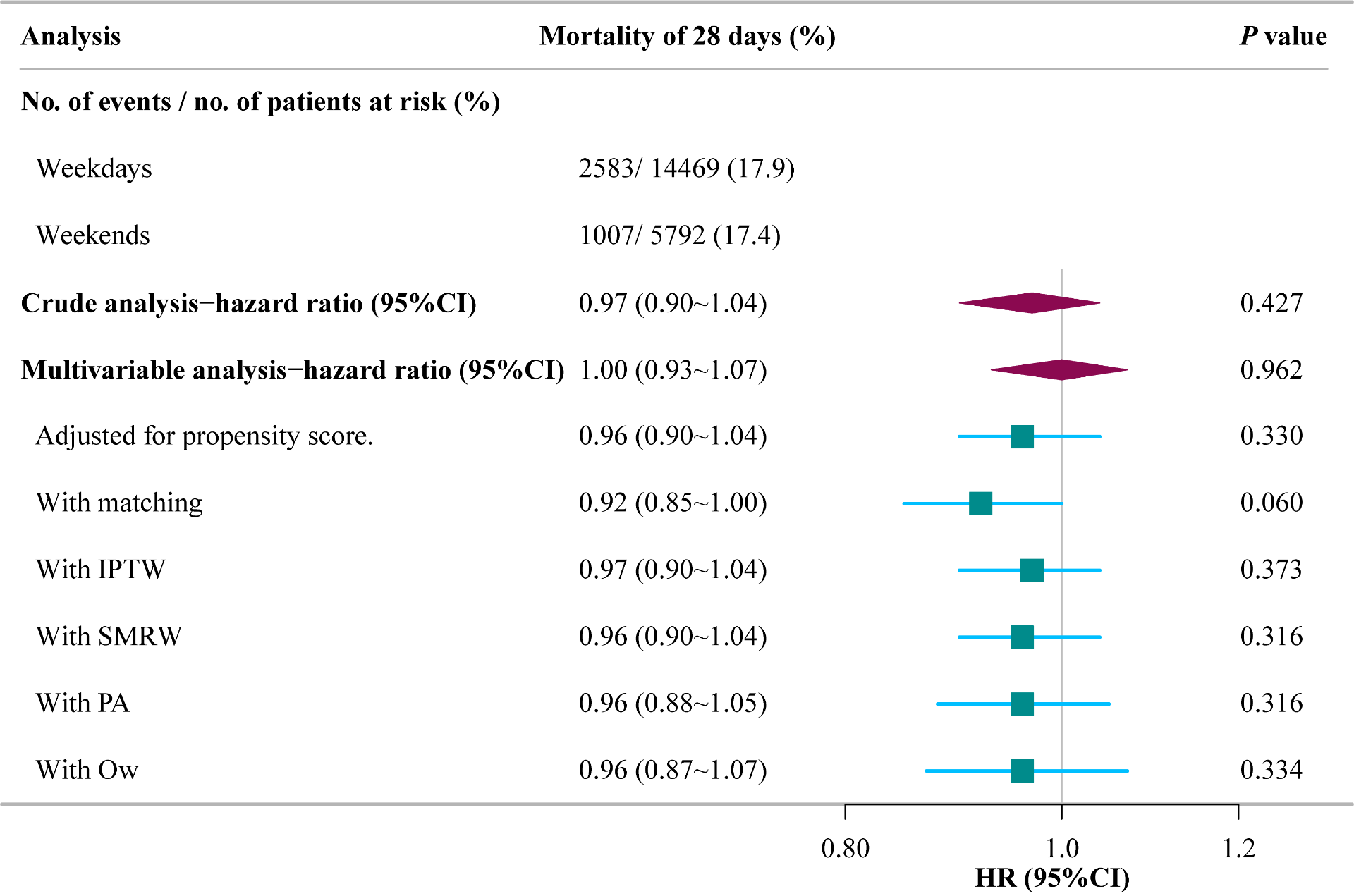

Supplement: S1 Fig — (TIF) [file pone.0324288.s005.tif]
